# RWD-derived response in multiple myeloma

**DOI:** 10.1371/journal.pone.0285125

**Published:** 2023-05-11

**Authors:** Tao Xu, James Roose, Mellissa Williamson, Ahmed Sawas, Wan-Jen Hong, Huan Jin, Kathleen Maignan, Alberto Rocci, Kasra Yousefi, Shaji Kumar, Stefka Tyanova

**Affiliations:** 1 F. Hoffmann-La Roche Ltd, Basel, Switzerland; 2 Genentech, Inc., South San Francisco, California, United States of America; 3 Flatiron Health, Inc., New York, New York, United States of America; 4 Mayo Clinic, Rochester, Minnesota, United States of America; Chinese Academy of Sciences, CHINA

## Abstract

Real-world data (RWD) are important for understanding the treatment course and response patterns of patients with multiple myeloma. This exploratory pilot study establishes a way to reliably assess response from incomplete laboratory measurements captured in RWD. A rule-based algorithm, adapted from International Myeloma Working Group response criteria, was used to derive response using RWD. This derived response (dR) algorithm was assessed using data from the phase III BELLINI trial, comparing the number of responders and non-responders assigned by independent review committee (IRC) versus the dR algorithm. To simulate a real-world scenario with missing data, a sensitivity analysis was conducted whereby available laboratory measurements in the dataset were artificially reduced. Associations between dR and overall survival were evaluated at 1) individual level and 2) treatment level in a real-world patient cohort obtained from a nationwide electronic health record-derived de-identified database. The algorithm’s assignment of responders was highly concordant with that of the IRC (Cohen’s Kappa 0.83) using the BELLINI data. The dR replicated the differences in overall response rate between the intervention and placebo arms reported in the trial (odds ratio 2.1 vs. 2.3 for IRC vs. dR assessment, respectively). Simulation of missing data in the sensitivity analysis (-50% of available laboratory measurements and -75% of urine monoclonal protein measurements) resulted in a minor reduction in the algorithm’s accuracy (Cohen’s Kappa 0.75). In the RWD cohort, dR was significantly associated with overall survival at all landmark times (hazard ratios 0.80–0.81, *p*<0.001) at the individual level, while the overall association was R^2^ = 0.67 (*p*<0.001) at the treatment level. This exploratory pilot study demonstrates the feasibility of deriving accurate response from RWD. With further confirmation in independent cohorts, the dR has the potential to be used as an endpoint in real-world studies and as a comparator in single-arm clinical trials.

## Introduction

Multiple myeloma (MM) is a bone marrow malignancy accounting for almost 10% of all haematologic cancers [1]. Nearly all patients with MM experience relapse after initial or salvage therapy [2]. Despite numerous advances in treatment options for MM, including the use of second-generation proteasome inhibitors (PIs) and immunomodulatory drugs (IMiDs), as well as antibody therapies [3], there is an unmet need for improved treatment and management options for patients with relapsed/refractory MM (RRMM) [4].

Assessment of patient response to therapy is an important element when determining appropriate treatments for MM [5, 6]. In 2006, the International Myeloma Working Group (IMWG) developed a set of response criteria that are commonly used by physicians in the assessment of patients with MM [7], which were further updated in 2016 [8]. These response criteria are based on the comparison of serial MM-specific laboratory measures, including levels of monoclonal (M) protein in the serum and urine, and serum free light chains (FLCs), as well as radiologic images and bone marrow investigations when appropriate.

The use of real-world data (RWD) is key to understanding the treatment course of patients with MM in clinical practice and the impact of novel treatments on all patients with MM, in particular those not eligible for or not reached by clinical trials. However, in routine clinical practice, the treatment responses recorded in patients’ medical records are often not standardized. Although some studies (in treated solid tumours) have shown that response rates can be successfully extracted from medical notes and radiologic reports stored in electronic health records (EHRs), they have also been reported to be overestimated [9, 10] and gaps within RWD impact the accuracy of response assessment. For example, bone marrow assessments are not routinely performed in the clinical setting due to their invasive nature and/or prohibitive cost. Moreover, M protein levels are not always measured or reported in both serum and urine [11].

To overcome the issue of missing data, we proposed a real-world derived response (dR) algorithm adapted from IMWG response criteria (‘flexible’ IMWG) that is able to estimate the response of patients with MM using less stringent criteria [12]. Here, in this exploratory pilot study, we further validate the dR algorithm and evaluate its accuracy and clinical utility using both clinical trial data and EHRs. Laboratory measurements collected from patients with RRMM in a phase III study (BELLINI; NCT02755597) [13] were used to test the dR algorithm in a clinical trial setting, whereby response evaluation by independent review committee (IRC) and the algorithm were compared. To demonstrate the utility of dR as a potential endpoint, we compared the accuracy of overall response rate estimates based on dR with those based on IRC response assessment. The association between dR and overall survival was also investigated in a real-world cohort of patients with MM obtained from the Flatiron Health MM database [14].

## Materials and methods

### Description of the dR algorithm

Flexible IMWG criteria, with exclusion of bone marrow biopsy data and imaging results, and reduction in either serum or urine M protein levels (but not both), were used to define the following dR categories: partial response (PR), very good PR (VGPR), complete response (CR) and stringent CR (sCR) (Table 1). PR was assigned if any of the following criteria were met: (i) a reduction of >50% in at least two consecutive measurements of serum M protein, given that the requirement for measurable disease was met for serum M protein; (ii) a reduction of >90% in at least two consecutive urine M protein measurements, given that the requirement for measurable disease was met for urine M protein; or (iii) a reduction of >50% in FLC difference in two consecutive measurements, if M protein was unmeasurable (or unavailable) in serum or urine. For patients meeting the requirement for measurable disease for both serum and urine M protein, VGPR was assigned if (i) serum and urine M protein were detectable by immunofixation but not on electrophoresis; or (ii) there was a >90% reduction in serum M protein plus urine M protein level of <100 mg/24 hours. CR was assigned in case of negative immunofixation on serum and urine (with no requirement for bone marrow assessment). sCR was assigned when the flexible criterion for CR was met, in addition to a normal FLC ratio.

**Table 1:** Criteria for a derived real-world response algorithm based on IMWG criteria.

Response	IMWG criteria	‘Flexible’ IMWG criteria (real-world response algorithm)
**sCR**	CR as defined below plus normal FLC ratio and absence of clonal cells in bone marrow by immunohistochemistry or immunofluorescence	CR as defined below plus normal FLC ratio
**CR**	Negative immunofixation on the serum and urine and disappearance of any soft tissue plasmacytomas and <5% plasma cells in BM	Negative immunofixation on the serum and urine
**VGPR**	Serum and urine M protein detectable by immunofixation but not on electrophoresis or >90% reduction in serum M protein plus urine M protein level <100 mg/24h	Serum and urine M protein detectable by immunofixation but not on electrophoresis or >90% reduction in serum M protein plus urine M protein level <100 mg/24h
**PR**	>50% reduction of serum M protein AND reduction in 24 hours urinary M protein by >90% or to <200 mg/24h; if the serum and urine M protein are unmeasurable, a >50% decrease in the difference between involved and uninvolved FLC levels is required in place of the M protein criteria	>50% reduction of serum M protein OR reduction in 24 hours urinary M protein by >90% or to <200 mg/24h.
	If serum and urine M protein are not measurable, and serum free light assay is also not measurable, >50% reduction in plasma cells is required in place of M protein, provided baseline BM plasma cell percentage was >30%	If the serum and urine M protein are unmeasurable, a >50% decrease in the difference between involved and uninvolved FLC levels is required in place of the M protein criteria
	In addition to the above listed criteria, if present at baseline, a >50% reduction in the size of soft tissue plasmacytomas is also required	

CR, complete response; FLC, free light chain; h, hours; IMWG, International Myeloma Working Group; M, monoclonal; PR, partial response; sCR, stringent complete response; VGPR, very good partial response.

To generalize the algorithm, we required at least two laboratory measurements per patient; one at baseline and one following treatment initiation. Baseline measurements were defined as laboratory measurements obtained no earlier than 60 days prior to and no later than 30 days after the therapy start date. Confirmed response required two consecutive laboratory measurements to meet the response criteria for the given level or for a deeper response level (e.g. VGPR confirmed a previously observed PR). The best confirmed response was used in the comparison with the IRC’s assessment.

In all cases when multiple distinct tests or test types were required to meet a criterion, a maximum time difference of 20 days was allowed between consecutive tests. For example, urine M protein measured or reported on two consecutive days would contribute to a single response evaluation at the same time point, while urine M protein and serum M protein measured 30 days apart would not be combined to assign VGPR. The 20-day interval was empirically chosen to ensure that no assessment time points were lost, while making sure that tests belonging to different assessment time points were not combined. No minimum time difference between consecutive tests was applied.

### Validation of the dR algorithm using BELLINI clinical trial data

BELLINI was a randomized 2:1, double-blind, multicentre, phase III trial that evaluated venetoclax or placebo in combination with bortezomib and dexamethasone in patients with RRMM [13]. Between 19^th^ of July 2016 and 31^st^ of October 2017, a total of 291 patients with MM were enrolled in the BELLINI trial, with 194 and 97 patients in the treatment and placebo arms, respectively. Baseline patient and disease characteristics are shown in S1 Table in S1 File.

Serum and urine M protein and FLC levels were assessed at baseline and at various time points during the trial (S2 Table in S1 File).

Response assessment was performed by both investigator and IRC in BELLINI, with the IRC assessment used as the ‘gold standard’ when the dR algorithm was applied.

The number of responders (defined as having a confirmed PR or better [≥PR]) versus non-responders assigned independently by IRC and by the dR algorithm was compared with Cohen’s Kappa statistic to estimate the level of concordance. To test if the assignment of responders by the dR algorithm could be used to replicate the efficacy conclusions of the BELLINI trial, Cochran-Mantel-Haenszel tests were used to calculate differences in overall response rate between the treatment and placebo arms of the trial, and were stratified based on the number of prior lines of therapy and previous PI treatment.

To test the utility of the dR algorithm with incomplete data, gaps were artificially introduced into the BELLINI trial dataset. Missing data were simulated by randomly excluding (i) 50% of all laboratory measurements, and (ii) 50% of serum M protein and FLC assessments and 75% of the available urine M protein measurements. The levels of data reduction were chosen to reflect the availability of laboratory measurements previously observed in RWD [12, 15].

Patients without sufficient laboratory measurements for response assessment according to the dR algorithm criteria were assigned as non-responders to make overall statistical results comparable with the original BELLINI trial results.

### RWD—Evaluating the association between dR and overall survival

The nationwide Flatiron Health EHR-derived de-identified database is a longitudinal database, comprising de-identified patient-level structured and unstructured data, curated via technology-enabled abstraction and subject to obligations to prevent re-identification and protect patient confidentiality [14, 16, 17]. Data from patients diagnosed with MM between 1^st^ of January 2011 and 31^st^ of January 2021 were abstracted from the Flatiron Health database, and patients with a baseline serum M protein or FLC measurement plus at least one additional measurement of the same type after treatment initiation were eligible for the current analysis. Baseline measurements were defined as laboratory measurements obtained no earlier than 60 days prior to and no later than 30 days after therapy start date. During the study period, the de-identified data originated from approximately 280 US cancer clinics (~800 sites of care). The majority of patients in the database are treated in community oncology settings; relative community/academic proportions may vary depending on study cohort.

Treatment regimens received by patients in the database were categorized as follows: chemotherapy+steroid, PI+steroid, IMiD+steroid, PI+IMiD+steroid, PI+chemotherapy+steroid, chemotherapy+IMiD+steroid, IMiD+monoclonal antibody+steroid, PI+monoclonal antibody+steroid.

**Fig 1:**
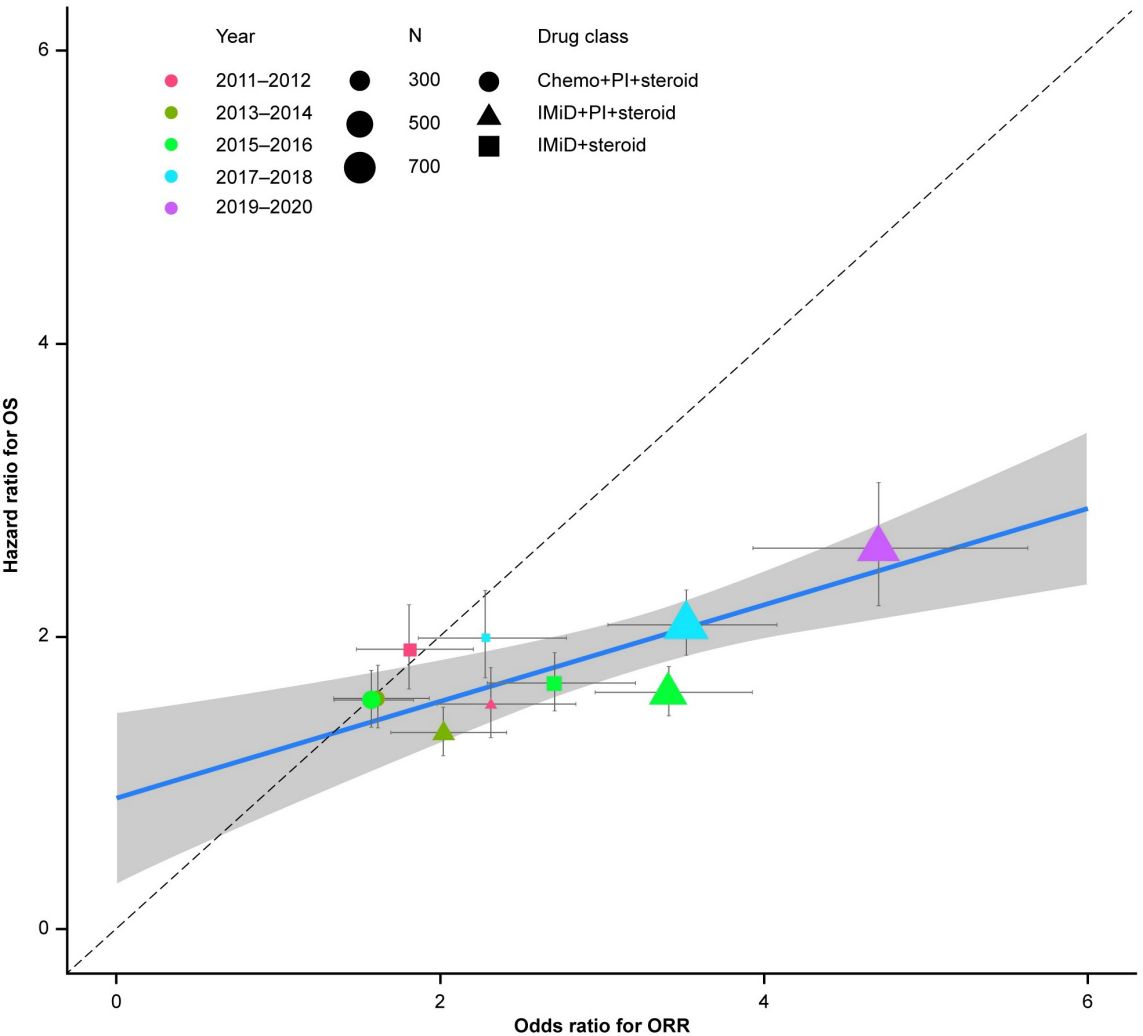
Study design. A) Landmark analysis for assessing individual-level associations and B) building cohorts for the assessment of treatment-level association between derived ORR and OS for a RWD cohort from the Flatiron Health MM database. MM, multiple myeloma; ORR, overall response rate; OS, overall survival; PD, progressive disease; RWD, real-world data.

Overall response (≥PR) to first-line treatment was derived for these patients using the dR algorithm. Associations between overall response rate and real-world overall survival were distinguished at both the individual and treatment level (Fig 1). At the individual level, landmark analyses were applied at approximately 3, 4 and 5 cycles of treatment, and again at 6 months (all patients were included in this analysis with no stratification by treatment). The hazard ratios (HRs) between patients with ≥PR and those not reaching PR by the landmark time were calculated based on a Cox proportional hazard model without adjustment. Patients who died before the landmark were excluded from the analysis. A treatment-level analysis was conducted to assess the association between comparative estimates of overall response rate and OS between different treatment groups over time. The cohort was stratified by year of treatment initiation (every 2 years from 2011–2020), and treatment groups in each 2-year stratum were compared to a reference group (PI+steroid) to estimate the HRs for overall survival and odds ratios (ORs) for the overall response rates determined by the dR algorithm using logistic regression and Cox proportional hazards models. Both measures of association were adjusted for potential confounding factors, including age, ECOG performance status, cytogenetic risk group (high vs. standard) and time between diagnosis and start of first-line treatment. Coefficient of determination (R^2^) from a stratum-size weighted linear regression model was used to assess the association between the resulting HRs and ORs, where a value close to 1 would imply a strong correlation and 0 would indicate no association [18]. Only significant HRs and ORs (*p*<0.05) were used in the treatment-level analysis.

## Results

### The dR algorithm response assessment is in concordance with that of the IRC

Data from all 291 patients enrolled in BELLINI were included in the current study. Concordance was high between the IRC’s and the dR algorithm’s assignments of responders classified as ≥PR (275/291 assignments in agreement, Cohen’s Kappa 0.83; Table 2).

**Table 2:** Number of responders (≥PR) and non-responders (<PR) in the BELLINI trial as assessed by IRC and the dR algorithm.

		**IRC assessment**		
		**Non-responder**	**Responder**	**All**
**dR algorithm assessment**	**Non-responder**	51	1	52
	**Responder**	15	224	239
	**All**	66	225	291

dR, derived response; IRC, independent review committee; PR, partial response.

In total there were 16 discrepant cases for which different responses were assigned by the IRC and the dR algorithm. In the only case assigned as a non-responder by the dR algorithm but as a PR by the IRC, there were not enough consecutive laboratory test results of the same type to confirm response, although other criteria were met. It is worth noting that the investigator in the BELLINI trial assessed this patient as having minimal response. Of the 15 cases in which the dR algorithm disagreed with the IRC assessment, eight were assigned as responders by investigator’s assessment, showing that there are cases in which the dR algorithm agrees more closely with the investigator’s assessment, and suggesting that these cases could be difficult to assess. The remaining 7/15 cases were assigned as responders by the dR algorithm as they met the criteria for reduction in either serum or urine M protein. It should be noted that agreement between investigator and IRC response assignment in BELLINI reached Cohen’s Kappa of 0.85, suggesting an upper limit to the attainable performance of the dR algorithm.

When depths of response were considered separately (i.e. PR, VGPR, CR and sCR) as opposed to grouping as ≥PR, concordance between IRC and the algorithm’s response assignments was lower (Cohen’s Kappa 0.56). This result was expected due to the omission of bone marrow data in the assessment of CR and sCR, which can cause the algorithm to over-assign VGPR as CR/sCR, and CR as sCR (S3 Table in S1 File).

### The dR algorithm reliably evaluates overall response rate and VGPR+ endpoints

The OR between the overall response rate (≥PR) of the intervention and placebo arms derived from the IRC response assessment in the BELLINI trial was 2.10 (overall response rate 82% [159/194] vs. 68% [66/97]), indicating the benefits of the treatment. The OR resulting from the dR algorithm assignment of overall response rate for the intervention versus the placebo arms was 2.31 (overall response rate 87% [168/194] vs. 73% [71/97]; Table 3).

**Table 3:** Analysis of the differences in overall response rate, ≥VGPR and ≥CR in the intervention and placebo arms of the BELLINI trial by IRC and the dR algorithm.

		Intervention arm	Placebo arm	Odds ratio	95% CI	CMH
		*N* = 194	*N* = 97			*p*-value*
**ORR**	**IRC**	159 (82%)	66 (68%)	2.10	1.21–3.67	0.0081
	**dR algorithm**	168 (87%)	71 (73%)	2.31	1.27–4.22	0.0053
**≥VGPR**	**IRC**	114 (59%)	35 (36%)	2.48	1.51–4.09	0.00029
	**dR algorithm**	116 (60%)	36 (37%)	2.49	1.51–4.10	0.00028
**≥CR**	**IRC**	51 (26%)	5 (5%)	6.47	2.49–16.78	0.00002
	**dR algorithm**	76 (39%)	22 (23%)	2.19	1.26–3.82	0.00528

*Nominal p-values without multiple hypotheses correction are shown for completeness and serve to compare the estimated treatment effect between dR and IRC.

CI, confidence interval; CMH, Cochran-Mantel-Haenszel; CR, complete response; dR, derived response; IRC, independent review committee; ORR, overall response rate; VGPR, very good partial response.

We then tested if the algorithm could be reliably used in the evaluation of other common endpoints such as ≥VGPR and ≥CR. Characterization of ≥VGPR by the algorithm was consistent with the IRC’s assessment (≥VGPR in intervention vs. placebo arms by IRC: 59% [114/194] vs. 36% [35/97], OR 2.48; by dR: 60% [116/194] vs. 37% [36/97], OR 2.49; Table 3). Characterization of ≥CR resulted in estimates of treatment efficacy that were lower, but directionally in agreement (≥CR in intervention vs. placebo arms by IRC: 26% [51/194] vs. 5% [5/97], OR 6.47; by dR: 39% [76/194] vs. 23% [22/97], OR 2.19; Table 3). This discrepancy can again be linked to the flexible IMWG criteria and the over-assignment of CR/sCR in the absence of bone marrow evaluation.

### Sensitivity analysis

The summary of the laboratory assessment frequency upon simulation of missing data can be seen in S4 Table in S1 File.

The original laboratory data contained two consecutive measurements of the same type for 283/291 patients (data missing for eight patients who discontinued the trial early) and thus confirmed response criteria were met for these patients. In line with the BELLINI trial, the analysis of which is based on the intention-to-treat population, we also included all patients and assigned the patients with insufficient laboratory tests as non-responders. Exclusion of those patients from the analysis resulted in a minor reduction of the Cohen’s Kappa statistic from 0.83 to 0.81 in the comparison of responders and non-responders between the dR algorithm and the IRC assessment (from 0.56 to 0.55 in the case of multiple depths of response). Upon reduction of 50% or 75% of the laboratory measurements, 277/291 patients were eligible for confirmed response criteria evaluation, with the remainder (14/291) automatically assigned as non-responders.

Randomly removing 50% of all laboratory measurements per patient resulted in a minor reduction in the algorithm’s accuracy in assessing the number of patients that exhibited partial response or better. In total 265 /291 assignments (54 non-responders and 211 responders) were in agreement with the IRC (Cohen’s Kappa 0.75; Table 4). The number of responders assigned by the dR algorithm decreased when missing data were introduced, as a second laboratory measurement that would have confirmed response was lost in some patients, thus leading to misclassification. As before, concordance decreased (Cohen’s Kappa 0.48) when depths of response were considered separately.

**Table 4:** Number of responders (≥PR) and non-responders (<PR) in the BELLINI trial as assessed by IRC and the dR algorithm using 50% of all laboratory measurements.

		**IRC assessment**		
		**Non-responders**	**Responders**	**All**
**dR algorithm assessment**	**Non-responders**	54	14	68
	**Responders**	12	211	223
	**All**	66	225	291

dR, derived response; IRC, independent review committee; PR, partial response.

Differences in overall response rate between the intervention and placebo arms in the trial based on IRC assessment could still be replicated by the algorithm after excluding 50% of all laboratory measurements (overall response rate in intervention vs. placebo arms by IRC: 82% [159/194] vs. 68% [66/97], OR 2.10; by dR: 80% [156/194] vs. 69% [67/97], OR 1.80; Table 5). Characterization of ≥VGPR and ≥CR by the algorithm resulted in estimates of treatment efficacy that were lower than by IRC (i.e. with lower OR), but directionally in agreement (≥VGPR in intervention vs. placebo arms by IRC: 59% [114/194] vs. 36% [35/97], OR 2.48; by dR: 41% [79/194] vs. 27% [26/97], OR 1.86; ≥CR in intervention vs. placebo arms by IRC: 26% [51/194] vs. 5% [5/97], OR 6.47; by dR: 21% [40/194] vs. 10% [10/97], OR 2.29; Table 5).

**Table 5:** Analysis of the differences in overall response rate, ≥VGPR and ≥CR in the intervention and placebo arms of the BELLINI trial by IRC and the dR algorithm using 50% of all available laboratory measurements.

		Intervention arm	Placebo arm	Odds ratio	95% CI	CMH
		(*N* = 194)	(*N* = 97)			*p*-value*
**ORR**	**IRC**	159 (82%)	66 (68%)	2.10	1.21–3.67	0.0081
	**dR algorithm**	156 (80%)	67 (69%)	1.80	1.04–3.12	0.0333
**≥VGPR**	**IRC**	114 (59%)	35 (36%)	2.48	1.51–4.09	2.9x10^-4^
	**dR algorithm**	79 (41%)	26 (27%)	1.86	1.09–3.16	0.0214
**≥CR**	**IRC**	51 (26%)	5 (5%)	6.47	2.49–16.78	2x10^-5^
	**dR algorithm**	40 (21%)	10 (10%)	2.29	1.08–4.86	0.0284

*Nominal p-values without multiple hypotheses correction are shown for completeness and serve to compare the estimated treatment effect between dR and IRC.

CI, confidence interval; CMH, Cochran-Mantel-Haenszel; CR, complete response; dR, derived response; IRC, independent review committee; ORR, overall response rate; VGPR, very good partial response.

Excluding 75% of urine M protein measurements, 50% of serum M protein, and 50% of FLC records per patient also resulted in a minor reduction in the algorithm’s accuracy in assessing the number of patients with ≥PR when compared with IRC assessment, with 265/291 assignments in agreement (Cohen’s Kappa 0.75; Table 6). Concordance decreased (Cohen’s Kappa 0.37) when depths of response were considered separately. The additional reduction of urine M protein measurements did not have a profound effect on the algorithm’s assessment, as the relaxed criteria only require a reduction in serum M protein for assignment of PR. The differences in overall response rate between the intervention and placebo arms in the trial based on IRC assessment were accurately replicated (overall response rate in intervention vs. placebo arms by IRC: 82% [159/194] vs. 68% [66/97], OR 2.10; by dR: 80% [155/194] vs. 68% [66/97], OR 1.83; Table 7). However, characterization of ≥VGPR and ≥CR by the algorithm resulted in estimates of treatment efficacy that were lower than those by IRC (≥VGPR in intervention vs. placebo arms by IRC: 59% [114/194] vs. 36% [35/97], OR 2.48; by dR: 27% [52/194] vs. 19% [18/97], OR 1.61; ≥CR in intervention vs. placebo arms by IRC: 26% [51/194] vs. 5% [5/97], OR 6.47; by dR: 10% [19/194] vs. 6% [6/97], OR 1.66; Table 7).

**Table 6:** Number of responders (≥PR) and non-responders (<PR) in the BELLINI trial as assessed by IRC and the dR algorithm using 50% of all laboratory measurements and 25% of all urine M protein measurements.

		**IRC assessment**		
		**Non-responders**	**Responders**	**All**
**dR algorithm assessment**	**Non-responders**	55	15	70
	**Responders**	11	210	221
	**All**	66	225	291

dR, derived response; IRC, independent review committee; M, monoclonal; PR, partial response.

**Table 7:** Efficacy analysis assessing differences in overall response rate, ≥VGPR and ≥CR in the intervention and placebo arms of the BELLINI trial by IRC and the dR algorithm using 50% of all available laboratory measurements and 25% of all urine M protein measurements.

		Intervention arm	Placebo arm	Odds ratio	95% CI	CMH
		(*N* = 194)	(*N* = 97)			*p*-value*
**ORR**	**IRC**	159 (82%)	66 (68%)	2.10	1.21–3.67	0.0081
	**dR algorithm**	155 (80%)	66 (68%)	1.83	1.06–3.16	0.0276
**≥VGPR**	**IRC**	114 (59%)	35 (36%)	2.48	1.51–4.09	2.9x10^-4^
	**dR algorithm**	52 (27%)	18 (19%)	1.61	0.88–2.96	0.1260
**≥CR**	**IRC**	51 (26%)	5 (5%)	6.47	2.49–16.78	2x10^-5^
	**dR algorithm**	19 (10%)	6 (6%)	1.66	0.63–4.38	0.3031

*Nominal p-values without multiple hypotheses correction are shown for completeness and serve to compare the estimated treatment effect between dR and IRC.

CI, confidence interval; CMH, Cochran-Mantel-Haenszel; CR, complete response; dR, derived response; IRC, independent review committee; M, monoclonal; ORR, overall response rate; VGPR, very good partial response.

For consistency, we tested the algorithm on the same datasets with missing data, but excluded the cases with insufficient laboratory measurements from the statistical analysis. The exclusion resulted in minor reduction of the Cohen’s Kappa statistic to 0.71 for both datasets with missing data when responders and non-responder assignments were compared and to 0.45 and 0.34 when multiple depths of response were considered in the 50% and 75% missing dataset, respectively.

#### Validation using RWD—Association between dR and OS

Of the 6,806 patients in the Flatiron Health MM database, 4,727 had valid laboratory test results for dR assessment during first-line treatment and were included in the study cohort. Baseline patient and disease characteristics are shown in S5 Table in S1 File. A total of 72% (3,387/4,727) were assigned as responders (i.e. ≥PR) by the dR algorithm. At the individual-level, dR (responder vs. non-responder) was significantly associated with overall survival (*p*<0.001) at all landmarks (3 cycles: HR 0.80 [95% CI, 0.72–0.88]; 4 cycles: HR 0.80 [95% CI, 0.72–0.89]; 5 cycles: HR 0.81 [95% CI, 0.72–0.90]; 6 months: HR 0.80 [95% CI, 0.72–0.90]) (Table 8). Subgroup analyses of responders versus non-responders showed a higher survival probability for responders at all landmarks (*p*<0.001 at cycles 3, 4 and 5, and at 6 months; S1 Fig). The most common treatment regimen for patients in the Flatiron Health MM database cohort was PI+IMiD+steroid (46% [2,188/4,727]), followed by PI+chemotherapy+steroid (14% [639/4,727]), PI+steroid (13% [621/4,727]), and IMiD+steroid (13% [607/4,727]); these four treatment groups accounted for 86% of all patients who were eligible for the treatment-level association analysis. The remaining treatment groups with fewer than 100 patients were not considered for analysis (S6 Table in S1 File).

**Table 8:** Landmark analysis of associations between dR and OS at 3rd, 4th, and 5th cycles and 6 months from start of first-line treatment in patients with MM.

	Non-responders (<PR)			Responders (PR+)				
Landmark time	*N*	Deaths	Median OS, months (95% CI)	*N*	Deaths	Median OS, months (95% CI)	HR (95% CI)	*p*-value
3 cycles^a^	1452	573	67.3 (60.6–80.0)	3071	968	79.6 (73.7–86.6)	0.80 (0.72–0.88)	1.7x10^-5^
4 cycles^a^	1314	516	66.3 (60.2–79.7)	3130	976	79.4 (73.9–91.0)	0.80 (0.72–0.89)	2.9x10^-5^
5 cycles^a^	1258	487	68.9 (61–79.4)	3123	966	79.8 (73.6–90.4)	0.81 (0.72–0.9)	1.0x10^-4^
6 months	1197	455	68 (60.1–80.0)	3072	934	80.7 (74.4–89.4)	0.80 (0.72–0.9)	1.4x10^-4^

The HRs between patients with PR or better and patients without response by the landmark time were calculated based on a Cox proportional hazard model without adjustment. Patients who died before the landmark were excluded from the analysis.

^a^28-day cycle.

CI, confidence interval; dR, derived response; HR, hazard ratio; MM, multiple myeloma; OS, overall survival; PR, partial response.

**Fig 2:**
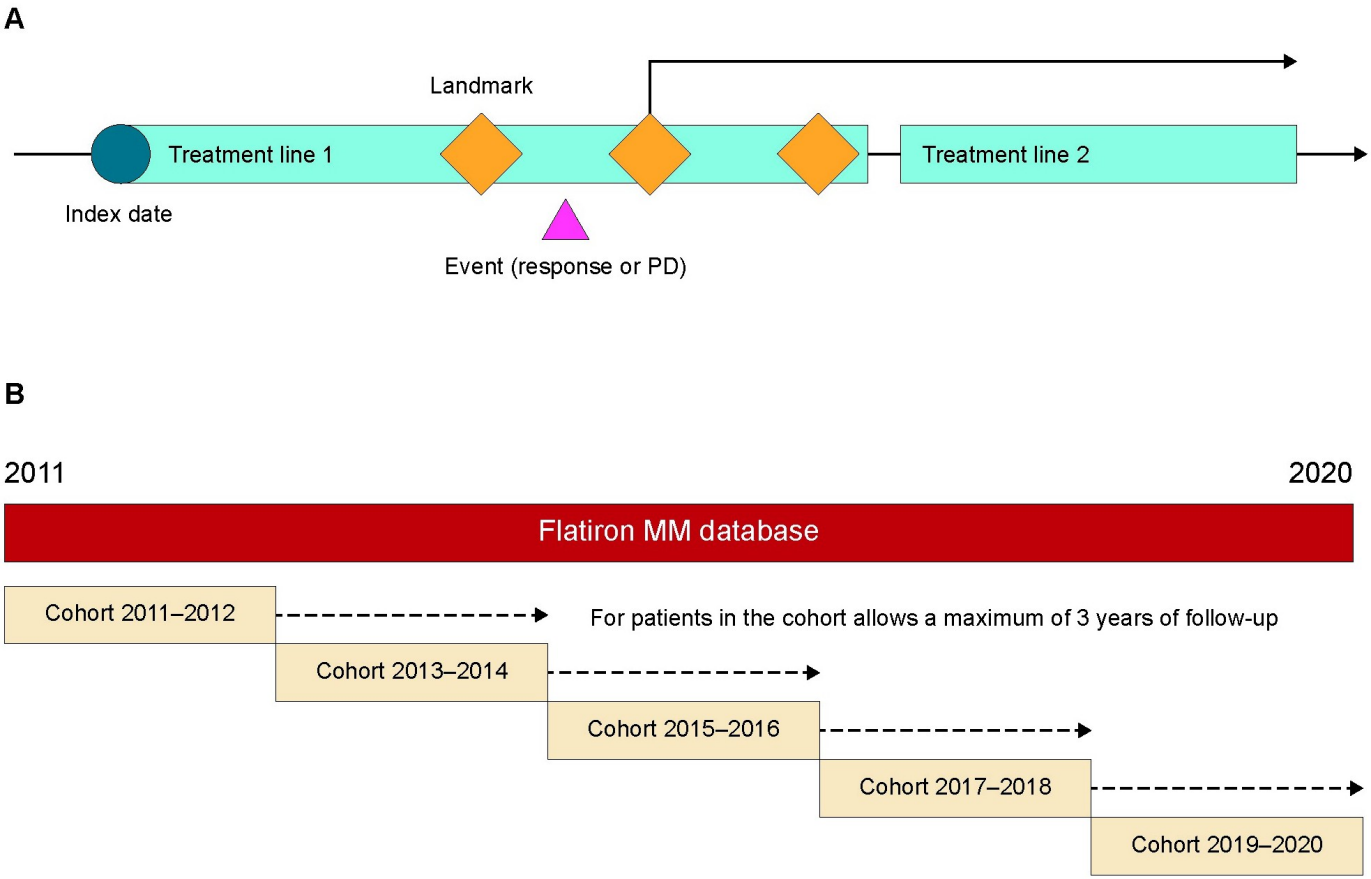
Treatment-level association between OR for derived overall response rate and HR for overall survival for a RWD cohort from the Flatiron Health MM database. Each dot in the plot represents one comparison in one cohort. For the treatment-level association analysis, only the significant ORs and HRs from the comparative analyses were used. PI+steroid was used as the comparator treatment group for all analyses. Error bars show the 95% CI of the estimated ORs and HRs. chemo, chemotherapy (cyclophosphamide, doxorubicin, melphalan, vincristine, bendamustine); CI, confidence interval; HR, hazard ratio; IMiD, immunomodulatory drug (lenalidomide, pomalidomide, thalidomide); MM, multiple myeloma; OR, odds ratio; ORR, overall response rate; OS, overall survival; PI; proteasome inhibitor (bortezomib, carfilzomib, or ixazomib); RWD, real-world data; steroid: dexamethasone, prednisone methylprednisolone, prednisolone.

The overall association between dR and overall survival had an R^2^ of 0.67 (*p*<0.001, Fig 2). For sub-group analysis by individual treatment group, only PI+IMiD+steroid versus PI+steroid had a sufficient sample size, and had an R^2^ of 0.82 (*p* = 0.02; S7 Table in S1 File). Thus, in the RWD cohort, dR was associated with overall survival at both an individual-level and at treatment-level.

## Discussion

Automated algorithms are increasingly used in clinical decision management due to their reliability and reproducibility, timely assessment of a large sample of patients, and adherence to recommended clinical practice [19]. Diverse applications of automated algorithms can be found for disease diagnosis [20], patient risk stratification [21], and prognostic scores [22, 23], and previous studies have demonstrated their potential for assessment of treatment response and disease progression [24, 25].

In this exploratory pilot study, we developed a dR algorithm to determine whether response assessment of patients with MM can be accurately performed using a limited set of laboratory measurements. The response assessments made using the dR algorithm showed strong agreement with the assessment of experienced clinicians (IRC) in a clinical trial setting. In addition, the robustness of the algorithm was demonstrated via a sensitivity analysis, with the exclusion of 75% of urine M protein measurements and 50% of other laboratory measurements resulting in only a minor reduction in the algorithm’s accuracy in assessing the number of patients with ≥PR (discordance observed in 26/291 patients, compared with 16/291 when using all measurements). This suggests that, in the clinical setting, where laboratory measurements are often reported at a lower frequency than in trials [26], the dR algorithm may be as accurate as clinician assessment in evaluating response to treatment. This is particularly relevant given the high rate of missing urine assessments in clinical trials and in clinical practice, most likely due to difficulties with collection technique and sample storage [15]. However, it should be noted that concordance between IRC and the algorithm’s response assignments decreased when individual depths of response were considered separately, with most misclassified cases being VGPR overestimated as CR or sCR due to the exclusion of bone marrow data from the flexible algorithm criteria.

If further validated within other clinical trial and/or RWD cohorts, the dR algorithm proposed here could be used as a robust tool to derive response status for patients with MM in RWD databases. It overcomes the limitations of EHR-captured responses, where large discrepancies between studies with regards to the criteria used for response determination have been reported [11]. For example, in EHR-based RWD studies, the reported response criteria are often time-unspecified or use unconventional criteria; thus, results are often not directly comparable. Furthermore, fewer than 50% of patients with MM in routine clinical practice are able to have their treatment response status calculated by the strict IMWG response criteria due to incomplete clinical data in their medical records [11, 26]. Notably the dR algorithm determined the response and progression status for 70% of patients captured in the Flatiron Health MM database through the application of laboratory assessments. Moreover, the dR algorithm provides an objective way of calculating response status in these RWD MM patients, enabling direct comparisons between different RWD datasets.

Using the dR algorithm we accurately reproduced the analysis of efficacy in the phase III BELLINI trial when considering overall response rate and ≥VGPR. This lays the foundations for dR to be used as a real-world endpoint in single-arm clinical studies to help accelerate drug development in MM. Recent guidance from the US Food and Drug Administration (FDA) recognizes the potential use of RWD as a comparator arm in an externally controlled trial [27]. Previous regulatory submissions to the FDA have included RWD from EHRs, claims, post-marketing safety reports and retrospective medical record reviews [28]. There are limitations associated with the use of RWD as an external control arm, such as inaccurate, incomplete or unclear data entries [29], small sample sizes, concerns over data quality and methodological issues [28], and the need to exclude unmeasured confounding and selection bias. However, the results from our study suggest that the dR algorithm is a step towards overcoming some of these problems, providing a means to determine responses in the absence of complete patient records and response data in the EHR and a framework to compare the dR algorithm to IRC assessment.

Overall survival is the standard endpoint used in oncology clinical trials to assess treatment efficacy of newly developed drugs. An attractive alternative to overall survival is overall response rate, which offers the possibility of earlier assessment with smaller cohort sizes, and is generally based on objective and quantitative assessment. One challenge raised by the FDA in relation to overall response rate is that it may not always relate well to overall survival [30]. Here, we showed that dR was associated with overall survival at both an individual- and treatment-level in a real-world cohort of patients with MM receiving first-line treatment, suggesting that overall response rate may be used to estimate survival benefits.

Although the algorithm was assessed in both a clinical trial and a large RWD cohort, some questions require further investigation. It is unclear whether certain treatment groups, the time stratification by two years, or the underlying mode of action of the therapies might have had an influence on the association between dR and overall survival. It is also possible that bias may have been introduced into the sensitivity analysis by assuming missing data occurs at random; in EHRs missingness is more often informative (i.e. the chance of missing data may be directly linked to the unobserved value itself [31]). Further validation of the algorithm using data from different real-world and clinical trial datasets with varying degrees of missing data should validate the generalisability of the algorithm and mitigate these concerns. Approximately 30% of patients in the real-world database did not have sufficient laboratory values for dR assessment in the first-line, and the removal of data for the sensitivity analysis resulted in a reduction in the number of patients being assigned as responders due to a lack of laboratory measurements. Further research and methodological consideration may be required to understand how best to analyse outcomes in real-world patients, and generalise results from patients with sufficient laboratory values for dR assessment to a broader population of patients, including those for whom sufficient laboratory values are not available.

Here, we have shown that our newly developed dR algorithm based on flexible IMWG criteria can consistently reproduce response assessments for patients with MM in a clinical trial setting, and can also assess response status for a real-world patient cohort. With further validation in other real-world and clinical trial populations and with other degrees and mechanisms of missing data, dR has the potential to be used as an endpoint in real-world studies and as an endpoint in external comparator cohorts in the clinical trial setting.

## Supporting information

S1 Fig Kaplan-Meier curve comparing responders (PR+; blue) and non-responders (<PR; red) at different landmark times. Time presented in months from the landmark to the patient’s death or censoring. PR, partial response.
(TIF)

S1 File (DOCX)

## Abbreviations

CI: confidence interval
CMH: Cochran-Mantel-Haenszel
CR: complete response
dR: derived response
ECOG: Eastern Cooperative Oncology Group
EHR: electronic health record
FDA: Food and Drug Administration
FLC: free light chain
h: hours
HR: hazard ratio
IFE: immunofixation
IMiD: immunomodulatory drugs
IMWG: International Myeloma Working Group
IRC: independent review committee
M: monoclonal
Max: maximum
Min: minimum
MM: multiple myeloma
MR: minimal response
OR: overall response
ORR: overall response rate
OS: overall survival
PI: proteasome inhibitors
PR: partial response
RRMM: relapsed refractory multiple myeloma
RWD: real-world data
sCR: stringent complete response
VGPR: very good partial response
Y: year
