# Supplementary figures and images for: RWD-derived response in multiple myeloma
Source: PLoS One. 2023 May 11;18(5):e0285125. doi: 10.1371/journal.pone.0285125 (PMC10174483; doi:10.1371/journal.pone.0285125)

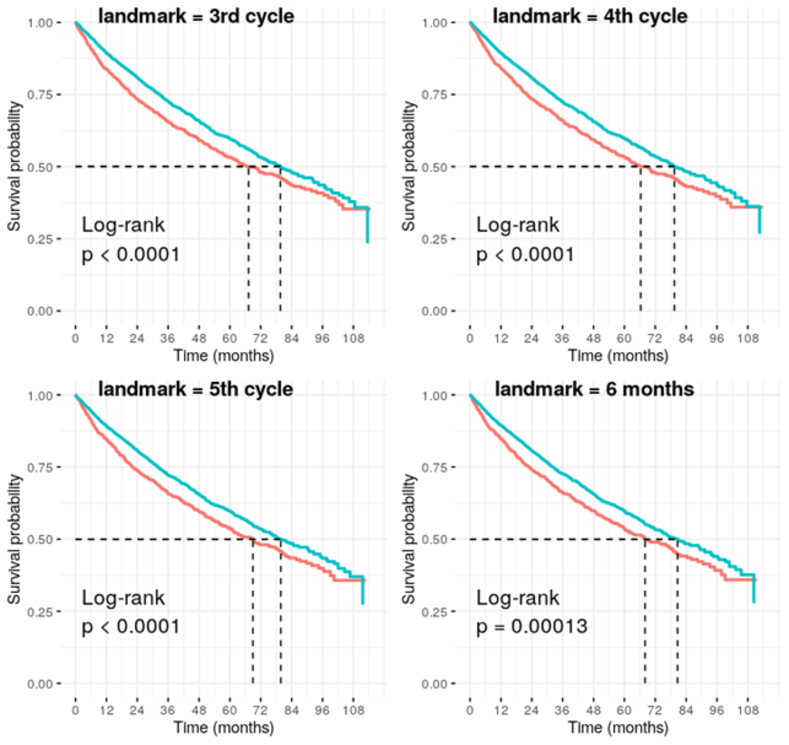

Supplement: S1 Fig — Time presented in months from the landmark to the patient’s death or censoring. PR, partial response. (TIF) [file pone.0285125.s001.tif]
