# Supplementary material for: RWD-derived response in multiple myeloma
Source: PLoS One. 2023 May 11;18(5):e0285125. doi: 10.1371/journal.pone.0285125 (PMC10174483; doi:10.1371/journal.pone.0285125)
Supplement: S1 File — (DOCX) [file pone.0285125.s002.docx]

**Supporting Information**

**S1 Table.** **Baseline patient and disease characteristics of patients with MM from the phase III BELLINI trial.**

|  | **Intervention arm (*n* = 194)** | **Placebo arm (*n* = 97)** |
| --- | --- | --- |
| **Median age, years (IQR)** | 66 (59–73) | 65 (61–71) |
| **Age 65 years** | 108 (56%) | 52 (54%) |
| **Sex**  Male  Female | 97 (50%)  97 (50%) | 55 (57%)  42 (43%) |
| **ISS stage at diagnosis**  Stage I  Stage II  Stage III  Not evaluable or missing data | 81 (42%)  69 (36%)  39 (20%)  5 (3%) | 48 (49%)  32 (33%)  13 (13%)  4 (4%) |
| **ECOG PS**  0  1 or 2  Missing | 101 (52%)  92 (47%)  1 (1%) | 47 (48%)  49 (51%)  1 (1%) |
| **Median time since diagnosis, years (IQR)** | 3.5 (2.1–5.8) | 4.0 (2.1–5.7) |
| **Number of previous lines of therapy**  1  2 or 3 | 91 (47%)  103 (53%) | 44 (45%)  53 (55%) |

ECOG PS, Eastern Cooperative Oncology Group performance status; IQR, interquartile range; ISS, international staging system; MM, multiple myeloma.

**S2 Table. Frequency and type of serum and urine laboratory tests from the BELLINI trial.**

| **Test type** | **Specimen** | **Test frequency per patient, median (range)** | **Total number of tests performed** |
| --- | --- | --- | --- |
| **FLC** | **Serum** | **13 (1–27)** | **3420** |
| **M protein** | **Serum** | **14 (1–27)** | **4010** |
|  | **Urine** | **12 (1–27)** | **3538** |
| **Immunofixation** | **Serum** | **5 (1–28)** | **2298** |
|  | **Urine** | **12 (1–28)** | **3429** |

FLC, free light chain; M, monoclonal; Max, maximum; Min, minimum.

**S3 Table. Agreement between response assignments by IRC and the dR algorithm.**

|  |  | **IRC assignment** | | | | | |
| --- | --- | --- | --- | --- | --- | --- | --- |
|  |  | **PR** | **VGPR** | **CR** | **sCR** | **Non-responder** | **Total** |
| **dR algorithm assignment** | **PR** | 72 | 8 | 0 | 0 | 7 | 87 |
|  | **VGPR** | 1 | 47 | 0 | 0 | 6 | 54 |
|  | **CR** | 1 | 26 | 2 | 0 | 1 | 30 |
|  | **sCR** | 1 | 12 | 37 | 17 | 1 | 68 |
|  | **Non-responder** | 1 | 0 | 0 | 0 | 51 | 52 |
|  | **Total** | 76 | 93 | 39 | 17 | 66 | 291 |

CR, complete response; dR, derived response; IRC, independent review committee; PR, partial response; sCR, stringent complete response; VGPR, very good partial response.

**S4 Table. Frequency and type of serum and urine laboratory tests used in the BELLINI trial when using 50% of all available laboratory measurements^a^.**

| **Test type** | **Specimen** | **Test frequency per patient, median (range)^b^** | **Total number of tests performed** |
| --- | --- | --- | --- |
| **FLC** | **Serum** | **6 (1–17)** | **1714** |
| **M protein** | **Serum** | **7 (1–17)** | **2005** |
|  | **Urine** | **6 (1–16)** | **1769** |
| **Immunofixation** | **Serum** | **4 (1–16)** | **1149** |
|  | **Urine** | **6 (1–16)** | **1715** |

^a^50% missing values have been simulated for serum and urine M protein, FLC and serum and urine IFE measurements.
^b^Patients with fewer than 1 laboratory measurement were filtered and did not contribute to the summary table.
FLC, free light chain; IFE, immunofixation; M, monoclonal; Max, maximum; Min, minimum.

**S5 Table.** **Baseline patient and disease characteristics of patients from the Flatiron Health MM database diagnosed with MM between 1^st^ of January 2011 and 31^st^ of January 2021 and included in the individual- and treatment-level analyses of associations between dR and OS.**

|  | **Overall**  **(*n* = 4727)** | **Non-responders (<PR)**  **(*n* = 1340)** | **Responders (≥PR)**  **(*n* = 3387)** |
| --- | --- | --- | --- |
| **Gender** |  |  |  |
| Female | 2183 (46.2%) | 615 (45.9%) | 1568 (46.3%) |
| Male | 2544 (53.8%) | 725 (54.1%) | 1819 (53.7%) |
| **Age** |  |  |  |
| Mean (SD) | 67.9 (10.5) | 68.3 (10.9) | 67.7 (10.3) |
| **ECOG PS** |  |  |  |
| 0 | 980 (20.7%) | 267 (19.9%) | 713 (21.1%) |
| 1 | 1046 (22.1%) | 260 (19.4%) | 786 (23.2%) |
| 2 | 390 (8.3%) | 114 (8.5%) | 276 (8.1%) |
| 3+ | 139 (2.9%) | 48 (3.6%) | 91 (2.7%) |
| Not known/missing | 2172 (45.9%) | 651 (48.6%) | 1521 (44.9%) |
| **ISS stage at diagnosis** |  |  |  |
| Stage I | 973 (20.6%) | 243 (18.1%) | 730 (21.6%) |
| Stage II | 994 (21.0%) | 251 (18.7%) | 743 (21.9%) |
| Stage III | 1046 (22.1%) | 295 (22.0%) | 751 (22.2%) |
| Unknown/not documented | 1714 (36.3%) | 551 (41.1%) | 1163 (34.3%) |
| **Follow-up time (months)** |  |  |  |
| Mean (SD) | 37.1 (27.6) | 34.6 (28.7) | 38.1 (27.0) |

dR, derived response; ECOG PS, Eastern Cooperative Oncology Group performance status; ISS, international staging system; MM, multiple myeloma; OS, overall survival; PR, partial response; SD, standard deviation.

**S6 Table. Size of each treatment group for the treatment-level association analysis.**

| **Year of first-line treatment initiation** | **Included/overall (*n*/*N*)** | **PI+chemo+steroid** | **PI+steroid** | **PI+IMiD+steroid** | **IMiD+steroid** |
| --- | --- | --- | --- | --- | --- |
| **2011–2012** | 368/486 | 47 (9.7%) | 95 (19.6%) | 113 (23.3%) | 113 (23.3%)3 |
| **2013–2014** | 676/809 | 158 (19.5%) | 139 (17.2%) | 225 (27.81%) | 154 (19.0%) |
| **2015–2016** | 980/1119 | 203 (18.1%) | 177 (15.8%) | 442 (39.5%) | 158 (14.1%) |
| **2017–2018** | 1097/1251 | 139 (11.1%) | 131 (10.5%) | 723 (57.79%)8 | 104 (8.3%) |
| **2019–2020** | 934/1062 | 92 (8.7%) | 79 (7.4%) | 685 (64.5%) | 78 (7.3%) |
| **Total** | 4055/4727 | 639 (13.5%) | 621 (13.1%) | 2188 (46.3%) | 607 (12.8%) |

chemo, chemotherapy; IMiD, immunomodulatory drug; PI; proteasome inhibitor.

**S7 Table. OR of dR and HR of OS between patients receiving different classes of treatment.**

|  |  | **OR of dR** | | **HR of OS** | |
| --- | --- | --- | --- | --- | --- |
| **Cohort** | **Treatment regimen** | **OR (95% CI)** | ***p*-value** | **HR (95% CI)** | ***p*-value** |
| 2011–2012 | **PI+IMiD+steroid** | **2.31 (1.56–3.42)** | **2.71x10^-5^** | **1.54 (1.15–2.05)** | **3.51x10^-3^** |
|  | **IMiD+steroid** | **1.81 (1.24–2.65)** | **2.34x10^-3^** | **1.91 (1.43–2.56)** | **1.21x10^-5^** |
|  | **PI+chemo+steroid** | **1.76 (1.06–2.92)** | **0.03** | **1.34 (0.93–1.94)** | **0.11** |
| 2013–2014 | **PI+IMiD+steroid** | **2.02 (1.44–2.83)** | **3.97x10^-5^** | **1.34 (1.06–1.7)** | **0.14** |
|  | IMiD+steroid | 1.12 (0.8–1.57) | 0.49 | 1.24 (0.98–1.55) | 0.67 |
|  | **PI+chemo+steroid** | **1.61 (1.14–2.29)** | **6.93x10^-3^** | **1.58 (1.22–2.03)** | **4.75x10^-4^** |
| 2015–2016 | **PI+IMiD+steroid** | **3.41 (2.59–4.49)** | **2.52x10^-18^** | **1.62 (1.33–1.96)** | **1.01x10^-6^** |
|  | **IMiD+steroid** | **2.71 (1.95–3.75)** | **2.11x10^-9^** | **1.68 (1.35–2.1)** | **4.23x10^-6^** |
|  | **PI+chemo+steroid** | **1.57 (1.17–2.12)** | **2.93x10^-3^** | **1.56 (1.23–1.98)** | **2.13x10^-4^** |
| 2017–2018 | **PI+IMiD+steroid** | **3.52 (2.64–4.69)** | **8.06x10^-18^** | **2.08 (1.7–2.56)** | **2.14x10^-12^** |
|  | **IMiD+steroid** | **2.28 (1.55–3.34)** | **2.78x10^-5^** | **1.99 (1.49–2.66)** | **2.59x10^-6^** |
|  | PI+chemo+steroid | 1.78 (1.19–2.64) | 4.60x10^-3^ | 1.17 (0.89–1.52) | 0.26 |
| 2019–2020 | **PI+IMiD+steroid** | **4.71 (3.32–6.67)** | **2.90x10^-18^** | **2.6 (1.91–3.55)** | **1.47x10^-9^** |
|  | IMiD+steroid | 1.3 (0.82–2.07) | 0.26 | 2.24 (1.43–3.51) | 4.64x10^-4^ |
|  | PI+chemo+steroid | 1.96 (1.25–3.07) | 3.57x10^-3^ | 1.29 (0.86–1.92) | 0.21 |

PI+steroid was used as the comparator group throughout. Multivariate analyses were adjusted for age, ECOG performance status, cytogenetic risk group (high vs. standard) and time from diagnosis to first-line treatment initiation. Significant results (*p*<0.05) used for the association analysis in Figure 2 are shown in bold.
chemo, chemotherapy; CI, confidence interval; dR, derived response; ECOG, Eastern Cooperative Oncology Group; HR, hazard ratio; IMiD, immunomodulatory drug; OR, odds ratio; OS, overall survival; PI; proteasome inhibitor.

**S1 Fig. Kaplan-Meier curve comparing responders (PR+; blue) and non-responders (<PR; red) at different landmark times.**

Time presented in months from the landmark to the patient’s death or censoring. PR, partial response.
